# Trends and Disparities in Mortality With Stroke as the Underlying Cause and Heart Failure as a Contributing Condition in the United States, 1999–2023

**DOI:** 10.1002/brb3.71753

**Published:** 2026-09-17

**Authors:** Ameen M. Bsharat, Ahmed A. Badawi, Aseel M. Shalalfeh, Diana A. Odeh, Murad J. Jaber, Deyaa A. Abd‐Alfattah, Obada R. Al‐Mashad, Mohammad M. Jayousi, Amjad S. Ibrahim

**Affiliations:** ^1^ Department of Medicine An‐Najah National University Nablus Palestine

**Keywords:** CDC WONDER, heart failure, mortality trends, stroke

## Abstract

**Background:**

National mortality studies have examined deaths in which stroke and heart failure (HF) are listed together, but trends specifically in deaths assigned to stroke as the underlying cause with HF recorded as a contributing condition remain less well defined.

**Methods:**

We analyzed CDC WONDER Multiple Cause of Death data for US decedents aged ≥15 years from 1999 to 2023. Stroke was defined by ICD‐10 codes I60–I69 as the underlying cause of death, and HF by code I50 as a contributing condition. Age‐adjusted mortality rates (AAMRs) per 100,000 population were assessed using Joinpoint regression. Analyses included sex, race, Hispanic origin, urbanization, and state. A sensitivity analysis restricted to 1999–2019, supplemented by AR(1) robustness analyses, evaluated whether the late‐period reversal preceded COVID‐19.

**Results:**

Among 170,303 qualifying deaths, AAMR declined from 4.09 in 1999 to 2.74 in 2023 (AAPC: −1.51%; p < 0.001). Mortality declined through 2009, remained stable during 2009–2016, and increased during 2016–2023 (APC: +5.38%). The pre‐pandemic sensitivity analysis confirmed a significant increase during 2014–2019 (APC: +3.04%; 95% CI: +2.06% to +4.77%; p < 0.001), which remained significant in AR(1) robustness analyses, including with positive autocorrelation fixed at ρ = 0.30 (APC: +3.02%; 95% CI: +1.01% to +8.02%; p = 0.020). The male‐to‐female AAMR ratio increased significantly during 2009–2023, despite nonsignificant terminal APCs within the male series. Among racial groups retained in the primary analysis, Black individuals had the highest AAMRs. AAMRs were higher in less urbanized areas, and state‐level AAMRs ranged from 1.05 in Arizona to 5.36 in Mississippi.

**Conclusions:**

Mortality with stroke as the underlying cause and HF as a contributing condition declined substantially before reversing in the mid‐2010s. Rates increased more sharply during the pandemic‐era years, superimposed on an increase that was already evident before 2020. Persistent racial, geographic, and urbanization disparities warrant continued surveillance and targeted prevention.

## Graphical abstract

Mortality in the United States with stroke as the underlying cause and heart failure as a contributing condition declined substantially from 1999 before reversing in the mid‐2010s. The increase was evident before the COVID‐19 pandemic and became more pronounced during 2020–2023. Persistent differences by race, Hispanic origin, urbanization, and geography were also observed, while sex‐specific patterns were more nuanced.

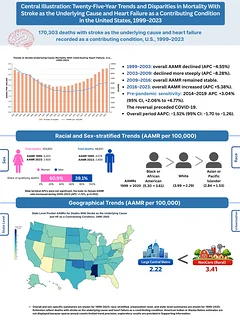

## 1 Introduction

Cardiovascular disease remains the leading cause of mortality in the United States and is a major contributor to stroke‐related morbidity and mortality. Heart failure affects more than 6 million US adults and confers a high risk of morbidity and premature mortality (Tsao et al. 2023). Recent national analyses have demonstrated a concerning plateau in the mid‐2010s after age‐adjusted cardiovascular mortality declined for several years (Sidney et al. 2016; Shah et al. 2019). Mortality rates therefore serve as critical indicators of population health and healthcare system performance. Given the shared pathophysiologic mechanisms between heart failure and stroke, as well as their frequent coexistence on death certificates, understanding population‐level mortality patterns for deaths in which stroke is the underlying cause and HF is recorded as a contributing condition is of substantial public health importance.

The risk of ischemic stroke is markedly elevated among patients with heart failure (Witt et al. 2006). Moreover, heart failure has been associated with higher post‐stroke mortality and recurrence compared with no heart failure (Pana et al. 2019). Proposed mechanisms linking HF to stroke risk and adverse cerebrovascular outcomes include impaired cardiac output, thromboembolic activation, intracardiac thrombus formation, atrial fibrillation, and structural or functional cardiac abnormalities (Doehner et al. 2023). Among older adults, the prevalence of heart failure and stroke escalates with advancing age (Benjamin et al. 2019). These clinical observations provide mechanistic context for why HF may be recorded as a contributing condition on death certificates assigning stroke as the underlying cause, particularly at older ages, while the present analysis remains a death‐certificate‐based population mortality study rather than a clinical cohort study of patients with HF.

Disparities have been documented across racial, sex‐based, and geographic groups in the United States (Howard et al. 2011; Carnethon et al. 2017; Pierce et al. 2025; Haast et al. 2012). Black individuals and residents of non‐urban areas experience higher stroke and cardiovascular mortality relative to White and urban individuals (Carnethon et al. 2017; Hammond et al. 2020; Pierce et al. 2025). Sex‐based disparities in stroke mortality have also been reported with evolving patterns over recent decades (Haast et al. 2012). Recent evidence also indicates that mortality patterns vary across sex and racial groups, although the direction and statistical strength of these differences depend on the outcome definition and analytic period. Non‐urban regions also exhibit elevated mortality rates with marked interstate variation. These patterns are consistent with multifactorial structural, socioeconomic, and healthcare‐access contexts that may be associated with observed differences among vulnerable populations.

A recent national analysis by Bilal et al. examined U.S. mortality among adults aged ≥25 years whose death certificates listed both stroke and HF, regardless of which condition was designated as the underlying cause of death (Bilal et al. 2026). That broad dual‐mention definition quantified the overall mortality burden in which the two conditions co‐occurred, but it combined distinct death‐certificate hierarchies, including deaths assigned primarily to stroke, HF, or another underlying condition. Because the underlying cause represents the condition selected as initiating the sequence leading to death, whereas multiple‐cause fields capture other reported conditions, these case definitions address different population‐surveillance questions. The prior study also incorporated additional hypertensive HF codes and selected stroke categories, whereas the present analysis required HF code I50 and used the complete I60–I69 range for stroke.

The present study therefore examined a narrower, directionally defined mortality phenotype: deaths for which stroke was assigned as the underlying cause and HF was recorded as a contributing condition. This approach focuses specifically on stroke‐attributed mortality with concomitant HF documentation rather than the broader population of deaths in which the two conditions were listed in any position. We aimed to characterize long‐term temporal trends and demographic and geographic disparities in this phenotype in the United States from 1999 to 2023. Secondary objectives included evaluating temporal changes in the male‐to‐female AAMR ratio and state‐level variation, which were not reported in the previous national analysis.

## 2 Methods

### 2.1 Study Design and Data Source

This was a population‐based serial cross‐sectional mortality study. Data were obtained from the CDC Wide‐ranging Online Data for Epidemiologic Research (CDC WONDER) Multiple Cause of Death (MCOD) database, which compiles US death certificate records and provides age‐adjusted mortality rates (AAMRs) with standard errors.

### 2.2 Study Population and Analytic Periods

We included decedents aged 15 years or older, applying CDC WONDER's 10‐year age‐group filters to enforce this restriction. Overall trends and analyses by sex and Hispanic origin were drawn from 1999 through 2023. Hispanic‐origin analyses were retained through 2023 because CDC WONDER provides the same Hispanic‐origin grouping under the selected Multiple Cause of Death query parameters across the full analytic period. For race‐stratified analyses, the period was capped at 2020, the last year for which CDC WONDER uses bridged‐race categories. From 2021 onward, the database shifts to a single‐race classification that is not interchangeable with the earlier scheme, so the two periods were kept separate rather than combined.

### 2.3 Case Definition and Cause‐of‐Death Hierarchy

Stroke was defined using ICD‐10 codes I60–I69 and was required to be assigned as the underlying cause of death. HF was defined using ICD‐10 code I50 and was required to appear in at least one additional multiple‐cause‐of‐death field on the same certificate. Because stroke was fixed as the underlying cause, HF represented a contributing condition in the analyzed records. Death certificates on which stroke appeared only as a contributing condition—including certificates assigning HF or another disease as the underlying cause—were not included.

This directionally defined case definition differs from a broad dual‐mention approach that includes any death certificate listing both stroke and HF regardless of the underlying‐cause assignment. The resulting outcome represents stroke‐underlying‐cause mortality with HF documented as a contributing condition. It should not be interpreted as all mortality involving coexisting stroke and HF, as mortality within a clinically confirmed HF cohort, or as evidence that HF independently caused the fatal stroke event.

### 2.4 Outcomes, Stratification, and Data Handling

The primary outcome was the AAMR for deaths in which stroke was assigned as the underlying cause and HF was recorded as a contributing condition, expressed per 100,000 persons in the general US population aged ≥15 years and standardized to the 2000 US standard population. Accordingly, the AAMRs represent population rates of this specific death‐certificate phenotype and were calculated using the general US population aged ≥15 years as the denominator, not the population of individuals living with stroke or HF. AAMRs and their standard errors were extracted directly from CDC WONDER. Mortality patterns were examined by sex, Hispanic origin, race, state, and urbanization category (2013 NCHS Urban‐Rural Classification Scheme). Race was analyzed independently of Hispanic origin. State‐level estimates were reported as pooled AAMRs for 1999–2020. Suppressed cells were not imputed and were excluded from calculations requiring exact counts.

### 2.5 Joinpoint Trend Analysis

Temporal trends were assessed using the National Cancer Institute Joinpoint Regression Program (version 5.4.0.0). Log‐transformed AAMRs were modeled as a function of calendar year because APC estimates quantify multiplicative, percentage‐based changes in rates over time and because log transformation is standard for estimating constant percentage change within each joinpoint segment. CDC WONDER‐derived standard errors were used for weighting, and models assumed uncorrelated errors. No separate formal tests of residual normality, homoscedasticity, or serial autocorrelation were performed. Accordingly, the uncorrelated‐error specification was treated as a modeling assumption rather than an empirically verified property of the primary and subgroup annual series. For the key pre‐pandemic sensitivity analysis, the potential influence of serial autocorrelation was additionally evaluated using first‐order autocorrelated error structures, as described below. The optimal number of joinpoints (0–4) was selected using the weighted Bayesian information criterion. The 0–4 joinpoint range was specified a priori and is consistent with common use of the National Cancer Institute Joinpoint program for annual mortality series of this length; models that selected the upper boundary were reviewed for clinical and epidemiologic face validity against the segment‐specific APC estimates. APC and AAPC estimates and their 95% confidence intervals were generated within the Joinpoint program using the empirical quantile method with 5,001 resamples. This was the prespecified confidence‐interval estimation procedure for the primary and subgroup models and was not conducted as a separate bootstrap sensitivity analysis. APC estimates were interpreted as the average annual percentage change within a segment, assuming a constant percentage change between adjacent joinpoints. Models were run for the full 1999–2023 series and all available subgroups. Because annual deaths among American Indian or Alaska Native individuals were sparse and the fitted Joinpoint model produced wide confidence intervals and a short three‐year segment, these subgroup estimates were treated as exploratory. They were therefore excluded from the primary figures and summary table and reported in Supporting Information S1, Supplementary Tables S4 and S8. No directional inference was based on the American Indian or Alaska Native segment‐specific APC estimates.

### 2.6 Pre‐Pandemic Sensitivity Analysis

To determine whether the late‐period mortality increase preceded the COVID‐19 pandemic, we repeated the overall Joinpoint analysis after restricting the time series to 1999–2019. The primary sensitivity analysis was conducted using Joinpoint Regression Program version 5.4.0.0, permitting zero to three joinpoints. The outcome definition, annual AAMRs, CDC WONDER‐derived standard errors, logarithmic transformation, uncorrelated‐error assumption, weighted Bayesian information criterion model selection, and empirical quantile confidence interval procedure were otherwise identical to those used in the primary analysis. As robustness checks, the sensitivity analysis was repeated using version 6.0.1 while permitting zero to three and zero to four joinpoints. All three specifications selected the same joinpoints and yielded identical APC, AAPC, confidence interval, p‐value, fitted‐value, SSE, and MSE results. To assess sensitivity to serial autocorrelation, additional analyses were conducted in Joinpoint version 6.0.1 using a first‐order autoregressive [AR(1)] error structure while retaining the CDC WONDER‐derived standard errors, logarithmic transformation, zero‐to‐three‐joinpoint range, weighted Bayesian information criterion model selection, and empirical quantile procedure with 5,001 resamples. The lag‐1 autocorrelation coefficient was first estimated from the data and the analysis was then repeated with fixed positive autocorrelation coefficients of ρ = 0.10, 0.20, and 0.30. The full‐period AAPC was calculated for 1999–2019, and the location and APC of the terminal segment were compared descriptively with those from the primary 1999–2023 model (Supplementary Methods S1).

### 2.7 Disparity Metrics and Geographic Visualization

A male‐to‐female AAMR ratio was computed annually to characterize relative sex differences over time, and formal inference on the rate‐ratio trajectory was performed using joinpoint regression. Sex‐specific APC models and the male‐to‐female rate‐ratio model addressed distinct questions: the sex‐specific models evaluated temporal change within each sex, whereas the rate‐ratio model evaluated change in the relative magnitude of male versus female AAMRs. On the logarithmic scale, the rate‐ratio model evaluates temporal change in the difference between log‐transformed male and female AAMRs, which represents a distinct null hypothesis with its own uncertainty structure from testing whether either sex‐specific APC differs from zero; therefore, statistical significance can differ between the ratio and component models without inconsistency. A significant rate‐ratio trend was not interpreted as evidence of a statistically significant increase within the male series or as a formal comparison of male and female APCs. Geographic variation was illustrated using a state‐level heatmap for 1999–2020. Urbanization and state‐level analyses were treated as descriptive pooled comparisons rather than annual Joinpoint series because smaller geographic strata are more susceptible to CDC WONDER suppression and unstable year‐to‐year rates.

### 2.8 Ethics

This study drew exclusively on publicly available, de‐identified mortality records obtained from CDC WONDER. Given the aggregated and non‐identifiable nature of these data, the study did not meet the definition of human subjects research, and institutional review board approval was not required.

## 3 Results

### 3.1 Overall Mortality Burden

**FIGURE 1:**
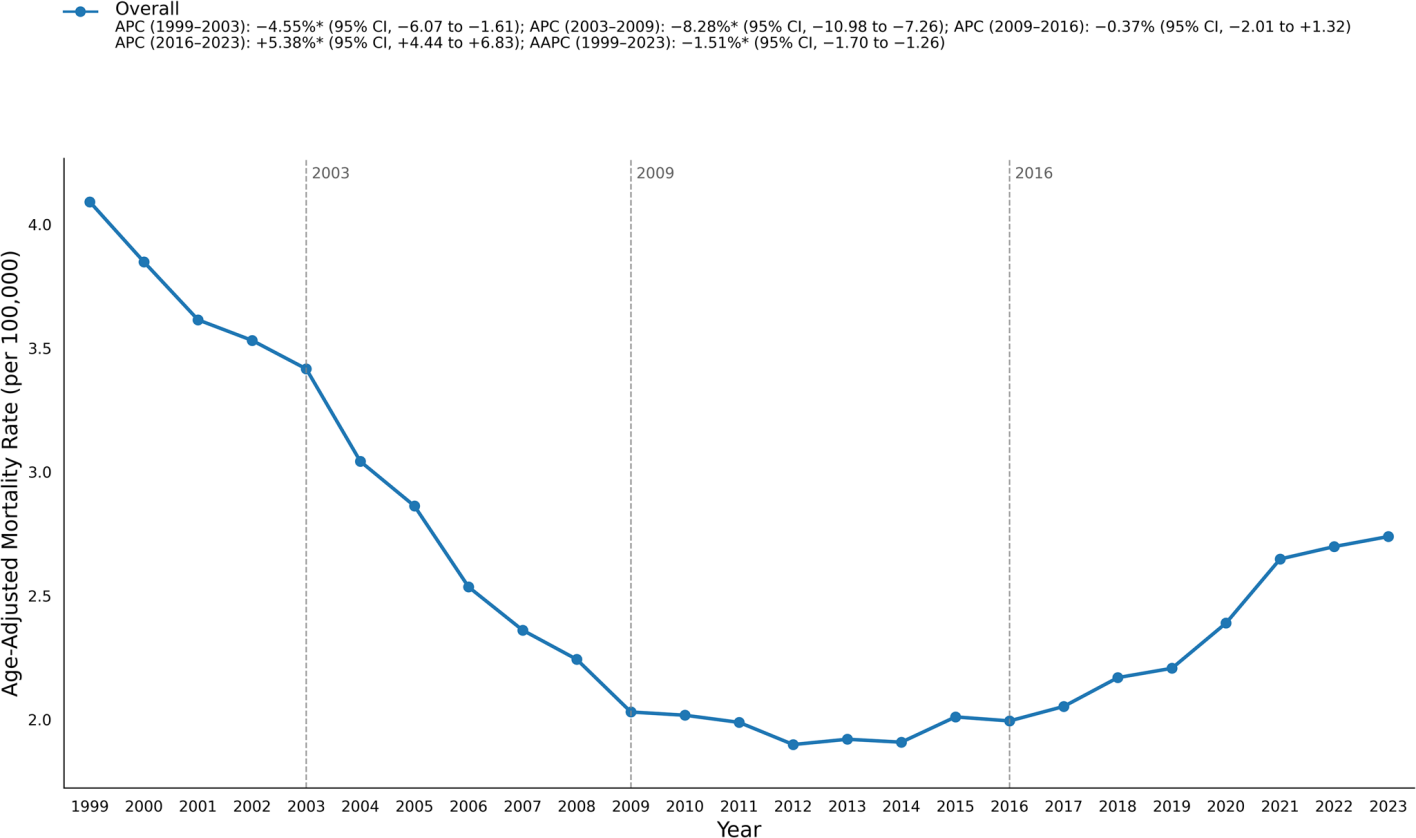
Trends in age‐adjusted mortality rates for deaths with stroke as the underlying cause and heart failure as a contributing condition, United States, 1999–2023.

A total of 170,303 deaths with stroke as the underlying cause of death and HF documented as a contributing condition were recorded in the United States between 1999 and 2023. Three joinpoints were identified in 2003 (95% CI: 2001–2006), 2009 (95% CI: 2008–2010), and 2016 (95% CI: 2015–2018). The AAMR declined from 4.09 per 100,000 persons aged ≥15 years in 1999 to 2.74 in 2023, reflecting an absolute reduction of 1.35 and a 33.0% relative decline. Mortality declined significantly from 4.09 in 1999 to 3.42 in 2003 (APC −4.55%; 95% CI: −6.07 to −1.61; p = 0.020), followed by a sharper reduction from 3.42 in 2003 to 2.03 in 2009 (APC −8.28%; 95% CI: −10.98 to −7.26; p = 0.006). From 2009 to 2016, mortality showed no significant change, changing from 2.03 in 2009 to 1.99 in 2016 (APC −0.37%; 95% CI: −2.01 to +1.32; p = 0.460), followed by a statistically significant increase from 1.99 in 2016 to 2.74 in 2023 (APC +5.38%; 95% CI: +4.44 to +6.83; p < 0.001). The lowest recorded AAMR was 1.90 in 2012; after reaching 2.21 in 2019, the AAMR increased to 2.39 in 2020 and 2.65 in 2021, corresponding to an absolute increase of 0.44 per 100,000 and a 20.0% relative increase from 2019 to 2021. The full‐period AAPC was −1.51% (95% CI: −1.70 to −1.26; p < 0.001) (Figure 1). A summary of selected Joinpoint APC and AAPC estimates across all modeled analyses is provided in Table 1.

**TABLE 1:** Summary of Joinpoint APC and AAPC estimates across primary and sensitivity analyses.

Analysis	Group	Years	APC (95% CI), p‐value	AAPC (95% CI), p‐value
Overall	Overall	1999–2003	−4.55 (−6.07 to −1.61) p = 0.020	−1.51 (−1.70 to −1.26) p < 0.001 (1999–2023)
		2003–2009	−8.28 (−10.98 to −7.26) p = 0.006	
		2009–2016	−0.37 (−2.01 to +1.32) p = 0.460	
		2016–2023	+5.38 (+4.44 to +6.83) p < 0.001	
Sensitivity analysis	Overall	1999–2003	−4.65 (−5.59 to −3.15), p < 0.001	−2.98 (−3.15 to −2.81) p < 0.001 (1999–2019)
		2003–2009	−8.06 (−9.47 to −7.42), p < 0.001	
		2009–2014	−1.21 (−3.23 to +0.22), p = 0.069	
		2014–2019	+3.04 (+2.06 to +4.77), p < 0.001	
Sex	Female	1999–2003	−4.04 (−5.34 to −1.17) p = 0.017	−1.63 (−1.83 to −1.41) p < 0.001 (1999–2023)
		2003–2007	−8.87 (−10.95 to −7.38) p < 0.001	
		2007–2012	−4.32 (−6.08 to −1.07) p = 0.024	
		2012–2018	+1.42 (−0.73 to +3.31) p = 0.110	
		2018–2023	+5.69 (+4.39 to +8.78) p = 0.002	
	Male	1999–2003	−4.50 (−5.91 to −1.46) p = 0.022	−1.37 (−1.59 to −1.09) p < 0.001 (1999–2023)
		2003–2009	−8.55 (−11.30 to −7.50) p < 0.001	
		2009–2016	+0.16 (−6.22 to +1.64) p = 0.773	
		2016–2021	+6.80 (−0.14 to +9.99) p = 0.059	
		2021–2023	+2.55 (−0.46 to +5.98) p = 0.065	
Rate Ratio	Male‐to‐Female Rate Ratio	1999–2009	−0.37 (−2.62 to +0.29) p = 0.249	+0.49 (+0.26 to +0.73) p < 0.001 (1999–2023)
		2009–2023	+1.10 (+0.73 to +2.49) p = 0.002	
Race	Asian or Pacific Islander	1999–2011	−7.09 (−8.58 to −5.92) p < 0.001	−3.11 (−3.61 to −2.55) p < 0.001 (1999–2020)
		2011–2020	+2.46 (+0.98 to +4.60) p = 0.002	
	Black or African American	1999–2011	−5.04 (−7.06 to −2.69) p = 0.033	−1.84 (−2.38 to −1.51) p < 0.001 (1999–2020)
		2011–2018	+0.90 (−6.91 to +2.58) p = 0.747	
		2018–2020	+8.72 (+1.85 to +12.90) p < 0.001	
	White	1999–2003	−4.53 (−5.92 to −1.47) p = 0.023	−2.69 (−2.93 to −2.38) p < 0.001 (1999–2020)
		2003–2009	−8.50 (−11.87 to −7.53) p < 0.001	
		2009–2014	−1.07 (−5.54 to +1.40) p = 0.283	
		2014–2020	+3.37 (+2.14 to +6.83) p = 0.005	
Hispanic Origin	Hispanic or Latino	1999–2012	−4.63 (−5.78 to −3.72) p < 0.001	−1.19 (−1.52 to −0.79) p < 0.001 (1999–2023)
		2012–2023	+3.03 (+2.15 to +4.18) p < 0.001	
	Not Hispanic or Latino	1999–2003	−4.43 (−6.14 to −1.02) p = 0.029	−1.49 (−1.71 to −1.22) p < 0.001 (1999–2023)
		2003–2009	−8.22 (−11.45 to −7.00) p = 0.007	
		2009–2015	−0.88 (−3.48 to +2.09) p = 0.328	
		2015–2023	+4.99 (+4.06 to +6.38) p = 0.005	

*Note*: The pre‐pandemic sensitivity analysis was restricted to 1999–2019. Its significant 2014–2019 APC indicates that the late‐period reversal was evident before the COVID‐19 pandemic; this increase remained significant in AR(1) robustness analyses, including with positive autocorrelation fixed at ρ = 0.30 (Supplementary Table S11). The positive male APC estimates for 2016–2021 and 2021–2023 were not statistically significant. APC magnitudes were not formally compared between males and females because the model‐selected segment boundaries differed by sex. American Indian or Alaska Native Joinpoint estimates are not included in the primary table because sparse annual deaths, wide confidence intervals, and a short three‐year segment limited reliable directional interpretation. Exploratory estimates are reported in Supporting Information.

Abbreviations: AAPC, average annual percent change; APC, annual percent change; CI, confidence interval.

### 3.2 Pre‐Pandemic Sensitivity Analysis

In the sensitivity analysis restricted to 1999–2019, Joinpoint regression identified inflection points in 2003 (95% CI: 2002–2005), 2009 (95% CI: 2008–2010), and 2014 (95% CI: 2012–2016). AAMR declined significantly from 1999 to 2003 (APC: −4.65%; 95% CI: −5.59% to −3.15%; p < 0.001) and from 2003 to 2009 (APC: −8.06%; 95% CI: −9.47% to −7.42%; p < 0.001). The change from 2009 to 2014 was not statistically significant (APC: −1.21%; 95% CI: −3.23% to +0.22%; p = 0.069). From 2014 to 2019, AAMR increased significantly by 3.04% per year (95% CI: +2.06% to +4.77%; p < 0.001). The 1999–2019 AAPC remained negative (−2.98%; 95% CI: −3.15% to −2.81%; p < 0.001), reflecting the larger declines earlier in the period. The pre‐pandemic increase was robust to serial‐autocorrelation correction. Under an AR(1) error model, the estimated lag‐1 autocorrelation coefficient was −0.165; the same joinpoints in 2003, 2009, and 2014 were selected; and the 2014–2019 increase remained significant (APC: +3.04%; 95% CI: +2.11% to +4.70%; p < 0.001). The same three joinpoints were retained when positive autocorrelation was fixed at ρ = 0.10, 0.20, and 0.30. The terminal increase remained statistically significant across all three specifications, including at ρ = 0.30 (APC: +3.02%; 95% CI: +1.01% to +8.02%; p = 0.020) (Supplementary Table S11). These findings demonstrate that the late‐period reversal was evident before the COVID‐19 pandemic. Because the final joinpoint was estimated within a 2012–2016 confidence interval, its onset is best interpreted as occurring during the mid‐2010s rather than in a single definitive year. This analysis indicates that the upward reversal was detectable in a series ending in 2019; it does not exclude an additional change in the level or trajectory of mortality during the pandemic period. The selected model and estimates were unchanged in robustness analyses conducted using Joinpoint version 6.0.1 while permitting up to three or four joinpoints. In the expanded zero‐to‐four‐joinpoint analysis, the three‐joinpoint model had lower BIC and Weighted BIC values than the four‐joinpoint candidate and therefore remained selected (Supplementary Figure S1; Supplementary Tables S9–11).

### 3.3 Sex‐Specific Trends

Analysis revealed four joinpoints for both sexes (females: 2003, 2007, 2012, 2018; males: 2003, 2009, 2016, 2021). Among females, AAMR declined significantly from 4.00 in 1999 to 3.38 in 2003 (APC −4.04%; 95% CI: −5.34 to −1.17; p = 0.017) and demonstrated a more pronounced decline from 3.38 in 2003 to 2.32 in 2007 (APC −8.87%; 95% CI: −10.95 to −7.38; p < 0.001), followed by a continued significant decline from 2.32 in 2007 to 1.86 in 2012 (APC −4.32%; 95% CI: −6.08 to −1.07; p = 0.024). A modest, non‐significant increase followed from 1.86 in 2012 to 2.04 in 2018 (APC +1.42%; 95% CI: −0.73 to +3.31; p = 0.110), and then a statistically significant increase from 2.04 in 2018 to 2.60 in 2023 (APC +5.69%; 95% CI: +4.39 to +8.78; p = 0.002). The full‐period AAPC was −1.63% (95% CI: −1.83 to −1.41; p < 0.001).

**FIGURE 2:**
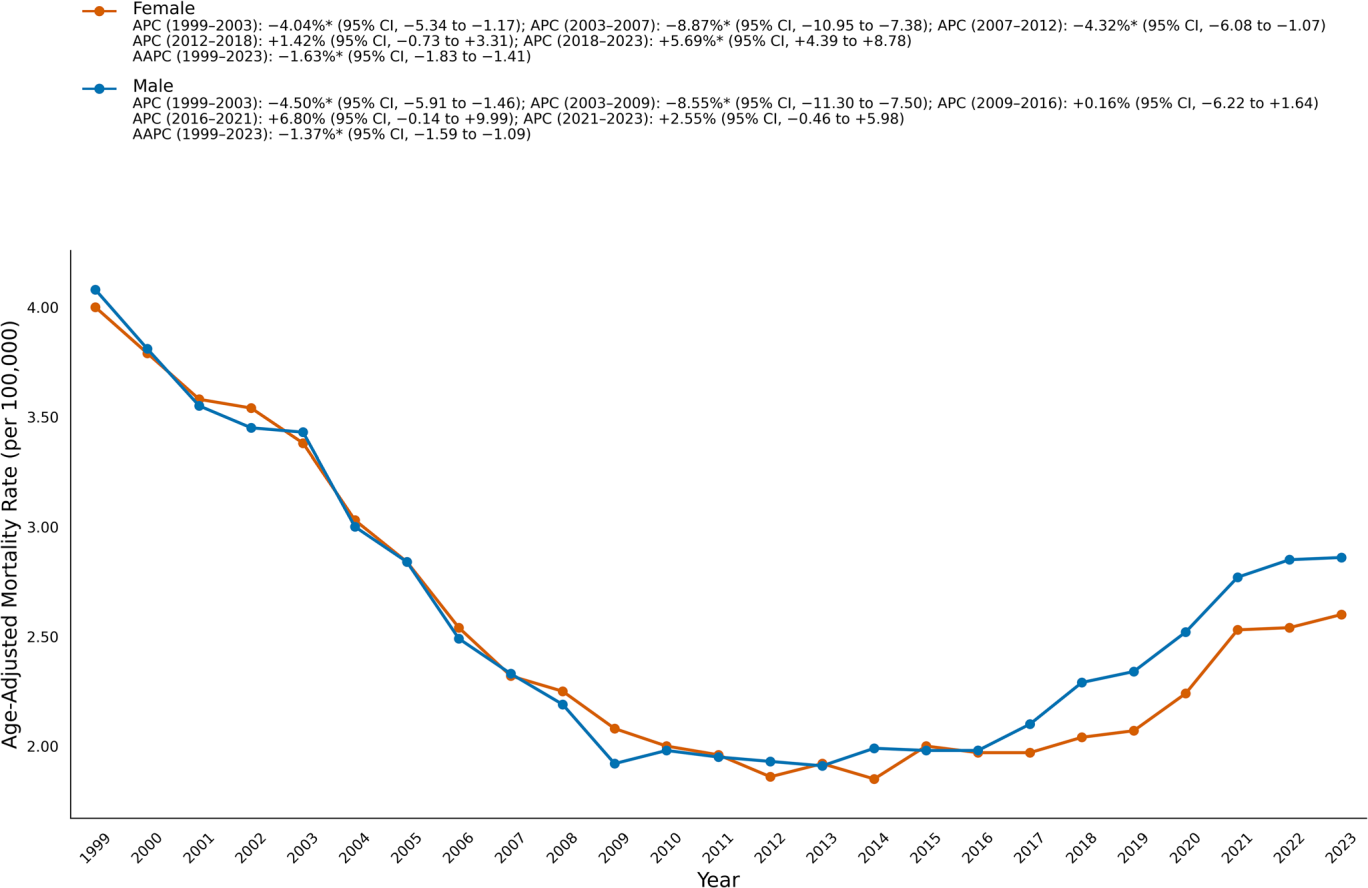
Sex‐stratified trends in age‐adjusted mortality rates for deaths with stroke as the underlying cause and heart failure as a contributing condition, United States, 1999–2023. The female AAMR increased significantly during 2018–2023, whereas the positive male APC estimates during 2016–2021 and 2021–2023 did not reach statistical significance.

Among males, AAMR declined significantly from 4.08 in 1999 to 3.43 in 2003 (APC −4.50%; 95% CI: −5.91 to −1.46; p = 0.022) and from 3.43 in 2003 to 1.92 in 2009 (APC −8.55%; 95% CI: −11.30 to −7.50; p < 0.001). From 2009 to 2016, AAMR was statistically stable, changing from 1.92 in 2009 to 1.98 in 2016 (APC +0.16%; 95% CI: −6.22 to +1.64; p = 0.773). Male AAMR increased numerically from 1.98 in 2016 to 2.77 in 2021 (APC: +6.80%; 95% CI: −0.14% to +9.99%; p = 0.059) and from 2.77 in 2021 to 2.86 in 2023 (APC: +2.55%; 95% CI: −0.46% to +5.98%; p = 0.065); however, neither terminal segment was statistically significant. The full‐period male AAPC was −1.37% (95% CI: −1.59% to −1.09%; p < 0.001). Male AAMRs were numerically higher than female AAMRs from 2017 onward, but this descriptive crossover should not be interpreted as evidence of a statistically significant late‐period increase within males (Figure 2). Annual sex‐specific counts and AAMRs are provided in Supporting Information S1, Supplementary Table S2.

### 3.4 Male‐to‐Female Mortality Rate Ratios

**FIGURE 3:**
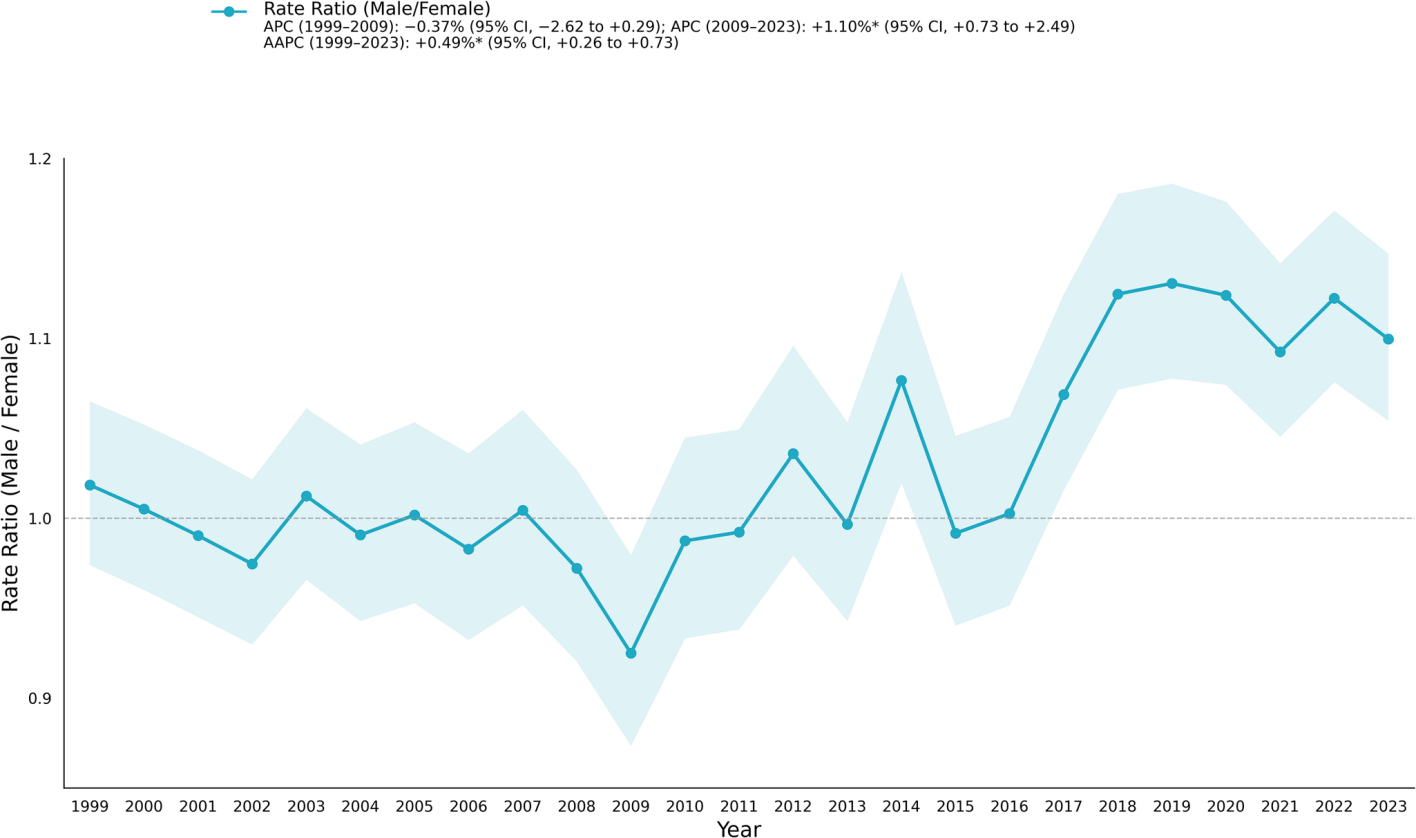
Trends in the male‐to‐female mortality rate ratio for deaths with stroke as the underlying cause and heart failure as a contributing condition, United States, 1999–2023. The shaded band represents the annual 95% confidence interval.

The male‐to‐female AAMR ratio showed one joinpoint in 2009 (95% CI for the joinpoint location, 2002–2018). The wide confidence interval for the joinpoint year indicates that the timing of the change was imprecisely estimated and should be interpreted as a gradual shift rather than an abrupt transition. From 1999 to 2009, the ratio changed from 1.018 to 0.925 without a statistically significant trend (APC: −0.37%; 95% CI: −2.62% to +0.29%; p = 0.249). From 2009 to 2023, the ratio increased significantly from 0.925 to 1.100 (APC: +1.10%; 95% CI: +0.73% to +2.49%; p = 0.002) (Figure 3).

Annual ratios were greater than 1.0, with 95% confidence intervals excluding 1.0, from 2017 through 2023; the ratio peaked at 1.131 (95% CI: 1.078–1.186) in 2019. The full‐period AAPC was +0.49% (95% CI: +0.26% to +0.73%; p < 0.001). These findings indicate a relative shift toward higher male than female AAMRs but do not establish a statistically significant late‐period increase within the male series. The significant rate‐ratio trend is not statistically inconsistent with the nonsignificant terminal male APCs because the ratio model evaluates change in the relative male‐to‐female trajectory rather than whether the male trajectory alone differs significantly from a zero slope. Annual ratios and confidence intervals are provided in Supporting Information S1, Supplementary Table S3.

### 3.5 Race‐Specific Trends

Race‐specific analyses were conducted through 2020 owing to data availability constraints. Annual deaths among American Indian or Alaska Native individuals were sparse, ranging from 15 to 48 per year, and the corresponding Joinpoint model yielded wide confidence intervals and a short three‐year segment. These estimates were therefore considered exploratory, omitted from the primary race‐specific figure and summary table, and reported in Supporting Information S1, Supplementary Tables S4 and S8. No directional conclusion was drawn from the American Indian or Alaska Native trend estimates.

**FIGURE 4:**
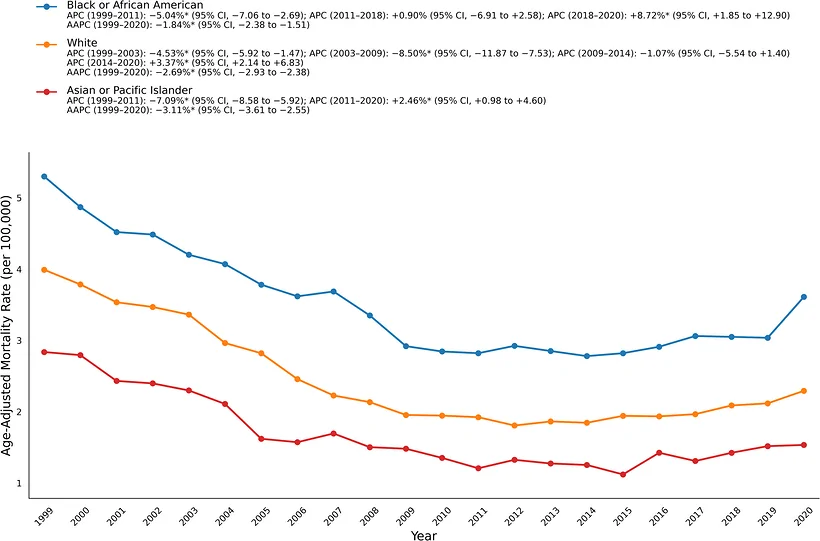
Race‐stratified trends in age‐adjusted mortality rates for deaths with stroke as the underlying cause and heart failure as a contributing condition, United States, 1999–2020. American Indian or Alaska Native estimates are reported in Supporting Information because sparse annual counts limited the precision of trend estimates.

Among Asian or Pacific Islander individuals, one joinpoint was identified in 2011. AAMR declined significantly from 2.84 in 1999 to 1.21 in 2011 (APC −7.09%; 95% CI: −8.58 to −5.92; p < 0.001), followed by a significant increase from 1.21 in 2011 to 1.53 in 2020 (APC +2.46%; 95% CI: +0.98 to +4.60; p = 0.002). The full‐period AAPC was −3.11% (95% CI: −3.61 to −2.55; p < 0.001). Among Black or African American individuals, two joinpoints were identified (2011, 2018). AAMR declined significantly from 5.30 in 1999 to 2.82 in 2011 (APC −5.04%; 95% CI: −7.06 to −2.69; p = 0.033), showed no significant change from 2.82 in 2011 to 3.05 in 2018 (APC +0.90%; 95% CI: −6.91 to +2.58; p = 0.747), and then increased sharply from 3.05 in 2018 to 3.61 in 2020 (APC +8.72%; 95% CI: +1.85 to +12.90; p < 0.001). The full‐period AAPC was −1.84% (95% CI: −2.38 to −1.51; p < 0.001). Because the 2018–2020 Black or African American segment includes only a short terminal window ending at the pandemic onset year, this APC should be interpreted as a short‐window descriptive estimate rather than evidence of a stable long‐term race‐specific trend. Among White individuals, three joinpoints were identified (2003, 2009, 2014). AAMR declined significantly from 3.99 in 1999 to 3.36 in 2003 (APC −4.53%; 95% CI: −5.92 to −1.47; p = 0.023) and from 3.36 in 2003 to 1.95 in 2009 (APC −8.50%; 95% CI: −11.87 to −7.53; p < 0.001), remained statistically stable from 1.95 in 2009 to 1.84 in 2014 (APC −1.07%; 95% CI: −5.54 to +1.40; p = 0.283), and then increased significantly from 1.84 in 2014 to 2.29 in 2020 (APC +3.37%; 95% CI: +2.14 to +6.83; p = 0.005). The full‐period AAPC was −2.69% (95% CI: −2.93 to −2.38; p < 0.001). Among the racial groups retained in the primary trend presentation, Black or African American individuals exhibited the highest AAMR throughout, while Asian or Pacific Islander individuals exhibited the lowest AAMR by 2020 (Figure 4). Annual race‐specific counts and AAMRs are provided in Supporting Information S1, Supplementary Table S4.

### 3.6 Mortality by Hispanic Origin

**FIGURE 5:**
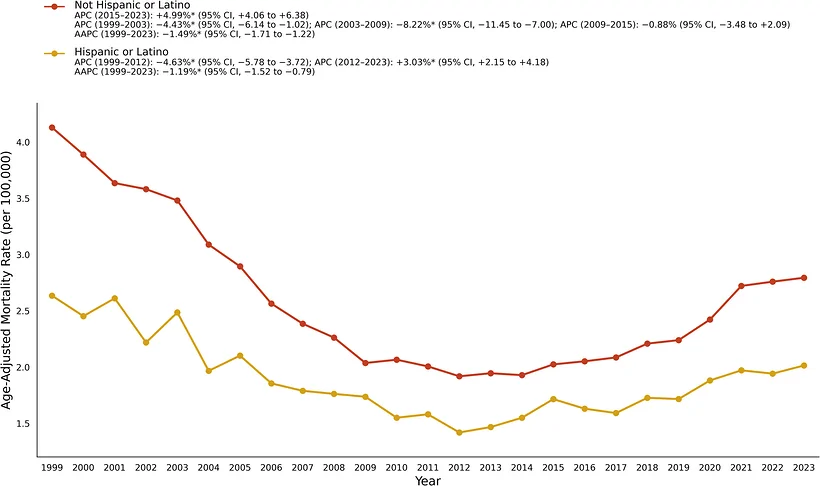
Trends in age‐adjusted mortality rates for deaths with stroke as the underlying cause and heart failure as a contributing condition by Hispanic origin, United States, 1999–2023.

Among Hispanic or Latino individuals, one joinpoint was identified in 2012. AAMR decreased markedly from 2.63 in 1999 to 1.42 in 2012 (APC −4.63%; 95% CI: −5.78 to −3.72; p < 0.001), followed by a significant increase from 1.42 in 2012 to 2.01 in 2023 (APC +3.03%; 95% CI: +2.15 to +4.18; p < 0.001). The full‐period AAPC was −1.19% (95% CI: −1.52 to −0.79; p < 0.001). Among non‐Hispanic or Latino individuals, three joinpoints were identified (2003, 2009, 2015). AAMR declined significantly from 4.13 in 1999 to 3.48 in 2003 (APC −4.43%; 95% CI: −6.14 to −1.02; p = 0.029), declined more sharply from 3.48 in 2003 to 2.04 in 2009 (APC −8.22%; 95% CI: −11.45 to −7.00; p = 0.007), showed no significant change from 2.04 in 2009 to 2.02 in 2015 (APC −0.88%; 95% CI: −3.48 to +2.09; p = 0.328), and then increased significantly from 2.02 in 2015 to 2.79 in 2023 (APC +4.99%; 95% CI: +4.06 to +6.38; p = 0.005). The full‐period AAPC was −1.49% (95% CI: −1.71 to −1.22; p < 0.001). Throughout the study period, non‐Hispanic or Latino individuals consistently exhibited higher AAMRs than Hispanic or Latino individuals (Figure 5). Annual Hispanic‐origin counts and AAMRs are provided in Supporting Information S1, Supplementary Table S5.

### 3.7 Urbanization‐Level Differences and State‐Level Variations

**FIGURE 6:**
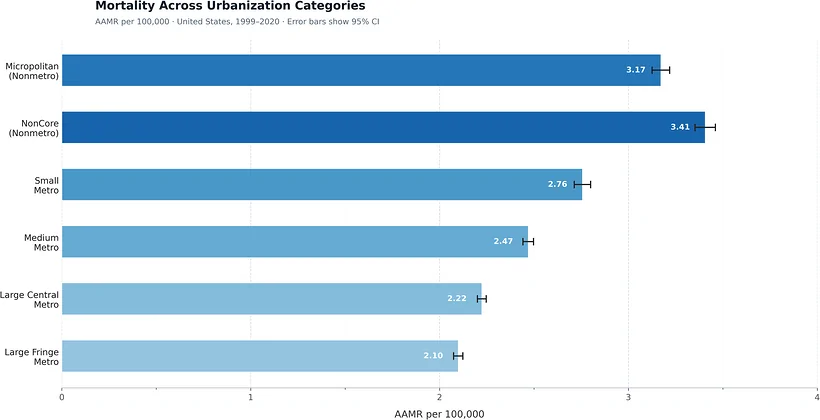
Age‐adjusted mortality rates for deaths with stroke as the underlying cause and heart failure as a contributing condition, by urbanization, United States, 1999–2020. Error bars represent 95% confidence intervals.

**FIGURE 7:**
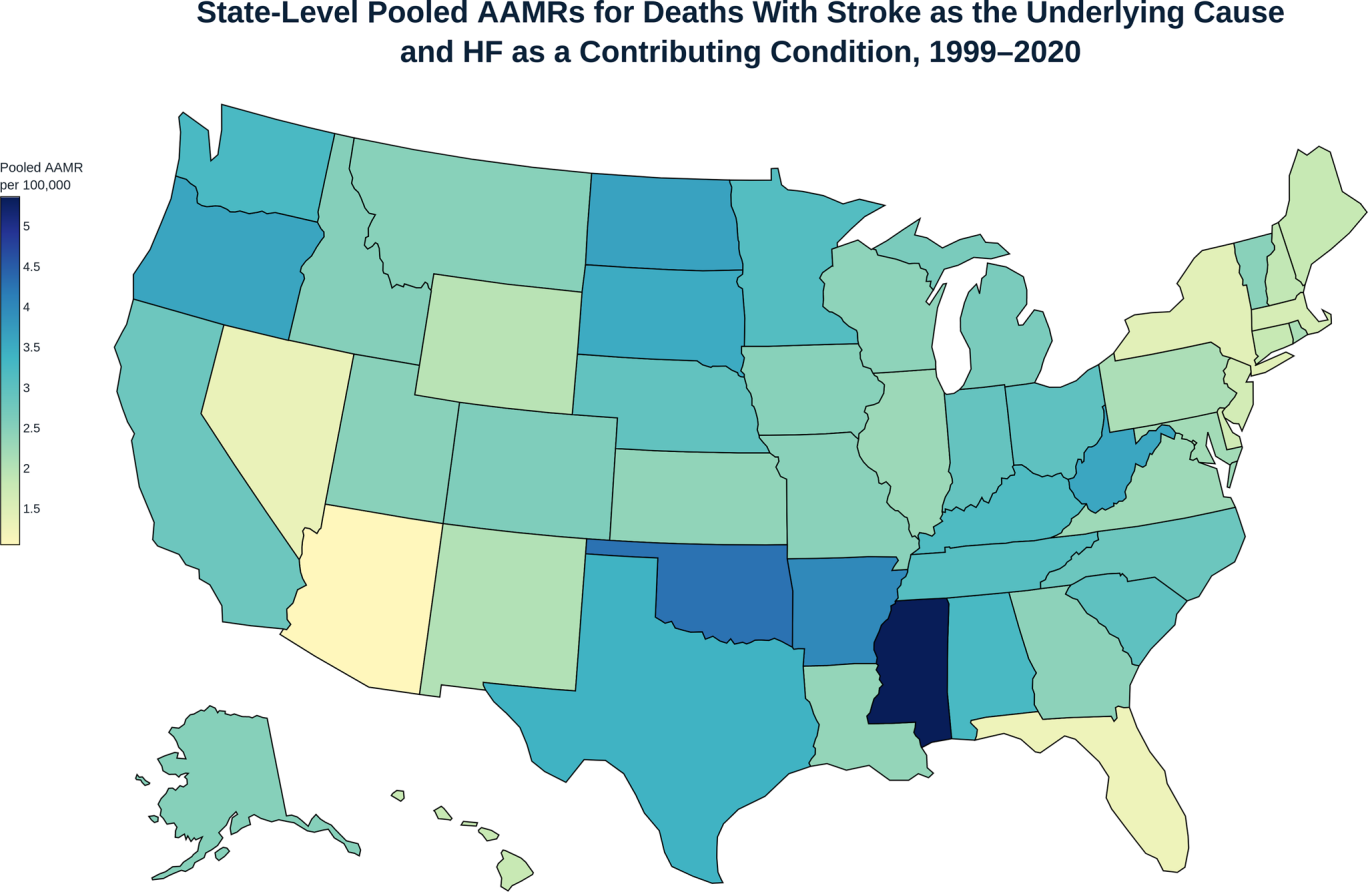
State‐level pooled age‐adjusted mortality rates for deaths with stroke as the underlying cause and heart failure as a contributing condition, United States, 1999–2020.

AAMRs varied by urbanization level during 1999–2020, with an inverse gradient observed. The highest AAMRs were seen in noncore nonmetropolitan areas (3.41 per 100,000) and micropolitan areas (3.17), followed by small metropolitan (2.76), medium metropolitan (2.47), large central metropolitan (2.22), and large fringe metropolitan areas (2.10) (Figure 6). During 1999–2020, state‐level AAMRs ranged from 1.05 per 100,000 in Arizona to 5.36 in Mississippi (Figure 7). Urbanization‐specific AAMRs are provided in Supporting Information S1, Supplementary Table S6, and state‐level AAMRs are provided in Supporting Information S1, Supplementary Table S7.

## 4 Discussion

This nationwide, population‐based analysis of 170,303 deaths with stroke assigned as the underlying cause and HF recorded as a contributing condition revealed several key findings. Overall AAMR declined substantially during the early study period, but this improvement was not sustained. Importantly, the pre‐pandemic sensitivity analysis identified a significant increase from 2014 to 2019, demonstrating that the reversal was already evident before the COVID‐19 pandemic. This finding remained statistically significant after accounting for first‐order serial autocorrelation: the data‐estimated lag‐1 correlation was −0.165, and the terminal increase remained significant even when positive autocorrelation was fixed at ρ = 0.30 (APC: +3.02%; 95% CI: +1.01% to +8.02%; p = 0.020). The primary 1999–2023 model identified a steeper terminal increase from 2016 to 2023, while AAMR rose markedly during 2020–2021 and remained elevated through 2023. Taken together, these findings indicate that the higher rates observed during 2020–2023 were superimposed on an increase that had already become statistically detectable before the pandemic.

These findings complement but are distinct from the recent analysis by Bilal et al., which identified 465,695 deaths among adults aged ≥25 years in whom HF and stroke were both recorded as multiple causes of death (Bilal et al. 2026). Their operational definition did not require stroke to be designated as the underlying cause and also used a different ICD‐10 code set, including I50, I11.0, I13.0, and I13.2 for HF and selected stroke categories. In contrast, our analysis required stroke codes I60–I69 to be assigned as the underlying cause and HF code I50 to be recorded as a contributing condition, identifying 170,303 qualifying deaths. The lower AAMRs observed in our study should not be attributed solely to the underlying‐cause restriction because age eligibility and ICD‐10 definitions also differed. Rather, the studies address complementary surveillance questions: Bilal et al. quantified the broader mortality burden represented by death certificates documenting both conditions, whereas the present study characterizes mortality assigned primarily to stroke when HF is additionally documented. Our findings demonstrate that the late‐period increase is also evident within this directionally restricted phenotype and additionally characterize male‐to‐female rate‐ratio trends and state‐level variation. The pre‐pandemic sensitivity analysis further extends this evidence by demonstrating that the increase within the underlying‐cause‐restricted phenotype was statistically detectable during 2014–2019, before the inclusion of pandemic‐era observations, and remained significant under AR(1) robustness analyses.

Prior literature has documented changes in cardiometabolic risk burden and cardiovascular prevention before 2020, as well as disruptions in cardiovascular and stroke care during the COVID‐19 pandemic (Lackland et al. 2014; Ritchey et al. 2020; Woodruff et al. 2024; Wu et al. 2020; Czeisler et al. 2020; Xie et al. 2022). These observations provide epidemiologic context for the temporal pattern but were not measured in the present study and should not be interpreted as explanations for the observed mortality changes.

Sex‐specific findings require cautious interpretation. Female AAMR increased significantly from 2018 to 2023, whereas the positive male APC estimates during 2016–2021 and 2021–2023 did not reach statistical significance. Separately, the male‐to‐female AAMR ratio increased significantly from 2009 to 2023, and annual ratios were above 1.0 from 2017 onward. These findings support a relative shift toward higher male than female AAMRs but do not demonstrate a statistically significant late‐period rise within males or a formally greater male slope. This apparent difference in statistical significance reflects the distinct hypotheses tested by the models: the rate‐ratio analysis evaluates change in the relative male‐versus‐female trajectory, whereas the sex‐specific models test temporal change within each sex separately. Previously reported sex differences in cardiovascular risk, healthcare use, and outcomes provide background context for the observed pattern (Rexrode et al. 2022; Poorthuis et al. 2017; Lam et al. 2019; Bushnell et al. 2014; Reeves et al. 2008; Lisabeth et al. 2009), but these factors were not measured in the present study.

Race‐stratified analyses demonstrated heterogeneity across groups, with Black individuals carrying the highest baseline AAMR. Prior literature has documented racial differences in hypertension burden, cardiovascular disease, socioeconomic conditions, healthcare access, and use of some preventive therapies (Ostchega et al. 2020; Triposkiadis et al. 2023; Forde et al. 2019; Reynolds et al. 2024). These factors provide background context only and were not evaluated as determinants of the observed race‐stratified AAMRs in the present study. The earlier declines occurred during a period in which improvements in hypertension management, heart failure treatment, and stroke care were reported nationally (Gorelick et al. 2020; Dubow and Fink 2011; Maddox et al. 2021; Centers for Disease Control and Prevention 2024); however, the present analysis cannot attribute the observed mortality changes to these developments. American Indian or Alaska Native annual trends were not interpreted in the primary analysis because sparse counts produced imprecise and unstable segment estimates.

The inverse urbanization gradient and state‐level variation parallel previously described U.S. rural mortality and Stroke Belt patterns (Liuzzo et al. 2024; Pierce et al. 2025). Geographic differences in stroke‐care availability and healthcare access have also been reported (Xian et al. 2011; Schieb et al. 2025; Turecamo et al. 2023; Hammond et al. 2020; Zhang et al. 2018), but these factors were not measured in the present study and are presented only as background context for the observed geographic differences.

### 4.1 Limitations

This study has several limitations. First, findings are based on death‐certificate data and ICD‐10 coding, which are subject to misclassification, incomplete recording of contributing conditions, and variation in reporting practices. In addition, designation of the underlying cause depends on the certifier's judgment and standardized coding rules; variation over time or across jurisdictions in assigning stroke, HF, or another condition as the underlying cause could influence the composition of this restricted mortality phenotype. Second, the outcome identifies deaths with stroke assigned as the underlying cause and HF recorded as a contributing condition on the death certificate; it does not define a cohort of patients with clinically confirmed HF, and the AAMR denominator is the general US population aged ≥15 years rather than the population with HF. Third, Joinpoint regression assumes constant percentage change within each modeled segment, and the log‐transformed AAMR model may be sensitive to small rates, sparse subgroup counts, standard‐error estimates, and selected joinpoint locations. Although the empirical quantile procedure does not itself correct serial autocorrelation, the key pre‐pandemic sensitivity analysis was additionally evaluated using AR(1) error structures; its 2014–2019 increase remained statistically significant both with the autocorrelation coefficient estimated from the data and with fixed positive coefficients from ρ = 0.10 to 0.30. The primary full‐period and subgroup models retained the prespecified uncorrelated‐error specification, so residual serial autocorrelation could still influence their standard errors and confidence intervals. Fourth, sex‐specific APCs and male‐to‐female rate‐ratio trends answer different statistical questions. The significant rate‐ratio trend indicates a change in relative male versus female AAMRs but does not establish a statistically significant increase within males or a formal difference between male and female APCs. These estimates should not be interpreted as independent causal effects. Fifth, not all analyses span the full 1999–2023 period: race‐stratified analyses were restricted to 1999–2020 owing to bridged‐race classification discontinuities. Sixth, American Indian or Alaska Native annual deaths were sparse, and the fitted joinpoint model produced wide confidence intervals and a short 2005–2008 segment. These estimates were therefore excluded from the primary presentation and retained only in Supporting Information as exploratory findings; they should not be interpreted as evidence of a stable directional trend. Seventh, the broad ICD‐10 stroke definition (I60–I69) includes multiple cerebrovascular categories and sequelae, which could not be separated in the primary death‐certificate phenotype. Eighth, the Hispanic and non‐Hispanic categories in CDC WONDER do not capture within‐group heterogeneity. Ninth, age‐stratified joinpoint models were not performed; although AAMRs were standardized to the 2000 US standard population, age standardization does not substitute for age‐specific trend analyses. Tenth, individual‐level variables, including socioeconomic status, healthcare access, and comorbidity burden, are unavailable. Eleventh, although the sensitivity analysis restricted to 1999–2019 demonstrated a significant pre‐pandemic increase that remained robust under AR(1) sensitivity analyses, the final joinpoint was estimated with a 95% CI spanning 2012–2016 and was followed by only five annual intervals through 2019. The precise onset of the reversal should therefore be interpreted as occurring during the mid‐2010s rather than in a single definitive year. In addition, the sensitivity analysis establishes that the reversal preceded 2020 but does not isolate or quantify an independent causal effect of the COVID‐19 pandemic. Finally, state‐level and urbanization analyses are area‐level ecological data and use pooled AAMRs rather than annual Joinpoint models because smaller geographic strata are more susceptible to suppression and unstable year‐to‐year rates; individual‐level inferences and ecological fallacy should therefore be considered when interpreting geographic patterns.

## 5 Conclusions

Between 1999 and 2023, mortality with stroke assigned as the underlying cause and HF recorded as a contributing condition declined substantially before reversing during the mid‐2010s. A sensitivity analysis restricted to 1999–2019, supported by AR(1) robustness analyses, confirmed a significant pre‐pandemic increase from 2014 to 2019, indicating that the reversal preceded COVID‐19. Rates increased more sharply during the pandemic‐era years, although the study was not designed to estimate an independent causal effect of the pandemic. Descriptive disparities were observed across race, Hispanic origin, urbanization, and geography. Sex‐specific results were more nuanced: female AAMR increased significantly during 2018–2023, whereas the positive terminal male APCs did not reach statistical significance, although the male‐to‐female AAMR ratio increased significantly after 2009. Because this death‐certificate‐based analysis cannot determine independent causal effects or clinical mechanisms, further individual‐level research is needed to clarify the clinical and health‐system factors associated with these observed population‐level trends. These findings characterize a specific cause‐of‐death hierarchy and should not be generalized to all deaths in which stroke and HF coexist or to mortality among patients with clinically confirmed HF.

## Author Contributions

**Ameen M. Bsharat**: conceptualization, methodology, data curation, formal analysis, investigation, Writing – original draft, Writing – review and editing, software. **Ahmed A. Badawi**: conceptualization, methodology, data curation, formal analysis, investigation, writing – original draft, writing – review and editing. **Aseel M. Shalalfeh**: visualization, writing – review and editing, writing – original draft. **Diana A. Odeh**: writing – original draft, writing – review and editing. **Murad J. Jaber**: writing – original draft, writing – review and editing. **Deyaa A. Abd–alfattah**: writing – original draft, writing – review and editing. **Obada R. Al–Mashad**: writing – original draft, writing – review and editing. **Mohammad M. Jayousi**: writing – original draft, writing – review and editing. **Amjad S. Ibrahim**: writing – original draft, writing – review and editing, conceptualization, methodology, data curation, formal analysis, investigation, supervision, project administration.

## Funding

The authors have nothing to report.

## Ethics Statement

This study used only publicly available, de‐identified, and aggregated national mortality data from the CDC WONDER Multiple Cause of Death database. Institutional review board approval was not required.

## Conflicts of Interest

The authors declare no conflicts of interest.

## Supporting information




**Supplementary Methods S1**; **Supplementary Tables S1–11**, containing annual mortality counts and rates, subgroup estimates, geographic estimates, exploratory American Indian or Alaska Native Joinpoint results, and pre‐pandemic sensitivity and robustness analyses; and **Supplementary Figure S1**.

## Data Availability

All data used in this study are publicly available. United States multiple‐cause‐of‐death mortality data (1999–2023) were obtained from the CDC WONDER Multiple Cause of Death database (https://wonder.cdc.gov/), accessed January 2026. Aggregated analysis‐ready annual series are available from the corresponding author upon reasonable request.
