# Supplementary material for: Trends and Disparities in Mortality With Stroke as the Underlying Cause and Heart Failure as a Contributing Condition in the United States, 1999–2023
Source: Brain Behav. 2026 Sep 17;16(9):e71753. doi: 10.1002/brb3.71753 (PMC13586657; doi:10.1002/brb3.71753)
Supplement: Supplementary file 1 [file BRB3-16-e71753-s001.docx]

Trends and Disparities in Mortality with Stroke as the Underlying Cause and Heart Failure as a Contributing Condition in the United States, 1999–2023

**Supporting Information S1**

**Supplementary Table 1. Annual overall mortality counts and rates for U.S. deaths with stroke as the underlying cause and heart failure as a contributing condition, 1999–2023.**

| **Year** | **Deaths** | **Population** | **Crude Rate** | **Age-Adjusted Rate** |
| --- | --- | --- | --- | --- |
| 1999 | 8,707 | 219,084,800 | 3.97426 | 4.09069 |
| 2000 | 8,317 | 221,168,531 | 3.76048 | 3.84824 |
| 2001 | 7,930 | 224,518,698 | 3.53200 | 3.61425 |
| 2002 | 7,875 | 227,062,163 | 3.46821 | 3.53109 |
| 2003 | 7,753 | 229,479,283 | 3.37852 | 3.41607 |
| 2004 | 7,002 | 232,153,496 | 3.01611 | 3.04238 |
| 2005 | 6,760 | 234,997,553 | 2.87663 | 2.86235 |
| 2006 | 6,110 | 237,863,203 | 2.56870 | 2.53529 |
| 2007 | 5,783 | 240,549,592 | 2.40408 | 2.36073 |
| 2008 | 5,630 | 243,186,582 | 2.31509 | 2.24280 |
| 2009 | 5,230 | 245,683,948 | 2.12875 | 2.03042 |
| 2010 | 5,290 | 247,518,325 | 2.13722 | 2.01724 |
| 2011 | 5,367 | 250,390,811 | 2.14345 | 1.98843 |
| 2012 | 5,260 | 252,769,942 | 2.08094 | 1.89889 |
| 2013 | 5,451 | 255,039,716 | 2.13731 | 1.92026 |
| 2014 | 5,556 | 257,789,101 | 2.15525 | 1.90845 |
| 2015 | 5,927 | 260,402,033 | 2.27610 | 2.01051 |
| 2016 | 6,058 | 262,152,444 | 2.31087 | 1.99432 |
| 2017 | 6,331 | 264,697,626 | 2.39179 | 2.05271 |
| 2018 | 6,889 | 266,281,990 | 2.58711 | 2.16917 |
| 2019 | 7,060 | 267,668,677 | 2.63759 | 2.20695 |
| 2020 | 7,807 | 269,190,697 | 2.90017 | 2.38937 |
| 2021 | 8,281 | 271,327,075 | 3.05204 | 2.64825 |
| 2022 | 8,918 | 273,850,170 | 3.25653 | 2.69841 |
| 2023 | 9,011 | 275,416,414 | 3.27177 | 2.73896 |

*Rates are expressed per 100,000 population.*

**Supplementary Table 2. Annual mortality counts and age-adjusted mortality rates by sex, 1999–2023.**

| **Year** | **Female** | | **Male** | |
| --- | --- | --- | --- | --- |
|  | **Deaths** | **AAMR** | **Deaths** | **AAMR** |
| 1999 | 5,626 | 4.00214 | 3,081 | 4.07586 |
| 2000 | 5,400 | 3.79388 | 2,917 | 3.81304 |
| 2001 | 5,153 | 3.58337 | 2,777 | 3.54865 |
| 2002 | 5,121 | 3.53532 | 2,754 | 3.44546 |
| 2003 | 4,952 | 3.38343 | 2,801 | 3.42518 |
| 2004 | 4,477 | 3.02710 | 2,525 | 2.99895 |
| 2005 | 4,282 | 2.83907 | 2,478 | 2.84401 |
| 2006 | 3,872 | 2.53587 | 2,238 | 2.49229 |
| 2007 | 3,638 | 2.32276 | 2,145 | 2.33298 |
| 2008 | 3,548 | 2.25232 | 2,082 | 2.18961 |
| 2009 | 3,350 | 2.07909 | 1,880 | 1.92296 |
| 2010 | 3,306 | 2.00065 | 1,984 | 1.97549 |
| 2011 | 3,330 | 1.96168 | 2,037 | 1.94636 |
| 2012 | 3,195 | 1.86290 | 2,065 | 1.92970 |
| 2013 | 3,330 | 1.92012 | 2,121 | 1.91344 |
| 2014 | 3,284 | 1.84972 | 2,272 | 1.99140 |
| 2015 | 3,599 | 2.00071 | 2,328 | 1.98393 |
| 2016 | 3,615 | 1.97311 | 2,443 | 1.97809 |
| 2017 | 3,679 | 1.96728 | 2,652 | 2.10255 |
| 2018 | 3,928 | 2.03840 | 2,961 | 2.29240 |
| 2019 | 3,979 | 2.07354 | 3,081 | 2.34419 |
| 2020 | 4,374 | 2.24158 | 3,433 | 2.51934 |
| 2021 | 4,620 | 2.53364 | 3,661 | 2.76784 |
| 2022 | 4,985 | 2.53759 | 3,933 | 2.84798 |
| 2023 | 5,009 | 2.59963 | 4,002 | 2.85863 |

*AAMR indicates age-adjusted mortality rate per 100,000 population.*

**Supplementary Table 3. Annual male-to-female age-adjusted mortality rate ratios, 1999–2023.**

| **Year** | **Male AAMR** | **Female AAMR** | **Rate Ratio** | **Lower 95% CI** | **Upper 95% CI** |
| --- | --- | --- | --- | --- | --- |
| 1999 | 4.07586 | 4.00214 | 1.018420 | 0.973983 | 1.064885 |
| 2000 | 3.81304 | 3.79388 | 1.005050 | 0.960140 | 1.052061 |
| 2001 | 3.54865 | 3.58337 | 0.990311 | 0.945055 | 1.037734 |
| 2002 | 3.44546 | 3.53532 | 0.974582 | 0.929823 | 1.021496 |
| 2003 | 3.42518 | 3.38343 | 1.012340 | 0.965867 | 1.061048 |
| 2004 | 2.99895 | 3.02710 | 0.990701 | 0.942898 | 1.040927 |
| 2005 | 2.84401 | 2.83907 | 1.001740 | 0.952750 | 1.053249 |
| 2006 | 2.49229 | 2.53587 | 0.982815 | 0.932347 | 1.036014 |
| 2007 | 2.33298 | 2.32276 | 1.004400 | 0.951629 | 1.060098 |
| 2008 | 2.18961 | 2.25232 | 0.972158 | 0.920374 | 1.026854 |
| 2009 | 1.92296 | 2.07909 | 0.924905 | 0.873417 | 0.979428 |
| 2010 | 1.97549 | 2.00065 | 0.987424 | 0.933217 | 1.044779 |
| 2011 | 1.94636 | 1.96168 | 0.992190 | 0.938182 | 1.049308 |
| 2012 | 1.92970 | 1.86290 | 1.035858 | 0.979229 | 1.095762 |
| 2013 | 1.91344 | 1.92012 | 0.996521 | 0.942913 | 1.053177 |
| 2014 | 1.99140 | 1.84972 | 1.076595 | 1.019625 | 1.136749 |
| 2015 | 1.98393 | 2.00071 | 0.991613 | 0.940305 | 1.045721 |
| 2016 | 1.97809 | 1.97311 | 1.002524 | 0.951492 | 1.056293 |
| 2017 | 2.10255 | 1.96728 | 1.068760 | 1.015683 | 1.124611 |
| 2018 | 2.29240 | 2.03840 | 1.124608 | 1.071552 | 1.180290 |
| 2019 | 2.34419 | 2.07354 | 1.130526 | 1.077687 | 1.185955 |
| 2020 | 2.51934 | 2.24158 | 1.123913 | 1.074159 | 1.175970 |
| 2021 | 2.76784 | 2.53364 | 1.092436 | 1.045312 | 1.141685 |
| 2022 | 2.84798 | 2.53759 | 1.122317 | 1.075657 | 1.171001 |
| 2023 | 2.85863 | 2.59963 | 1.099630 | 1.054251 | 1.146961 |

*AAMR indicates age-adjusted mortality rate per 100,000 population.*

Note: A rate ratio greater than 1.0 indicates a higher male than female AAMR in that year. Annual rate-ratio comparisons and the Joinpoint trend in the ratio do not imply a statistically significant temporal increase within the male series.

**Supplementary Table 4. Annual mortality counts and age-adjusted mortality rates by race, 1999–2020.**

| **Year** | **AIAN** | | **API** | | **Black** | | **White** | |
| --- | --- | --- | --- | --- | --- | --- | --- | --- |
|  | **Deaths** | **AAMR** | **Deaths** | **AAMR** | **Deaths** | **AAMR** | **Deaths** | **AAMR** |
| 1999 | 20 | 2.62931 | 114 | 2.83596 | 907 | 5.29890 | 7,666 | 3.98985 |
| 2000 | 21 | 2.39421 | 119 | 2.79292 | 834 | 4.86873 | 7,343 | 3.78417 |
| 2001 | 22 | 2.49212 | 113 | 2.43106 | 793 | 4.51856 | 7,002 | 3.53409 |
| 2002 | 21 | 2.26988 | 114 | 2.39634 | 798 | 4.48425 | 6,942 | 3.46806 |
| 2003 | 30 | 3.22494 | 122 | 2.29720 | 760 | 4.20047 | 6,841 | 3.36073 |
| 2004 | 29 | 2.69608 | 118 | 2.10772 | 743 | 4.06915 | 6,112 | 2.96261 |
| 2005 | 30 | 3.01487 | 101 | 1.61926 | 720 | 3.78097 | 5,909 | 2.81897 |
| 2006 | 23 | 2.16720 | 106 | 1.57154 | 708 | 3.61738 | 5,273 | 2.45646 |
| 2007 | 15 | 1.39816 | 117 | 1.69372 | 739 | 3.68516 | 4,912 | 2.22681 |
| 2008 | 19 | 1.61235 | 111 | 1.50171 | 702 | 3.34952 | 4,798 | 2.13360 |
| 2009 | 16 | 1.26948 | 114 | 1.47988 | 625 | 2.91845 | 4,475 | 1.95335 |
| 2010 | 16 | 1.29814 | 112 | 1.35113 | 624 | 2.84363 | 4,538 | 1.94434 |
| 2011 | 23 | 1.54306 | 109 | 1.20570 | 652 | 2.81965 | 4,583 | 1.92227 |
| 2012 | 17 | 1.10441 | 135 | 1.32442 | 693 | 2.92297 | 4,415 | 1.80543 |
| 2013 | 27 | 1.65607 | 134 | 1.27275 | 709 | 2.85036 | 4,581 | 1.86234 |
| 2014 | 20 | 1.22506 | 145 | 1.25248 | 710 | 2.77895 | 4,681 | 1.84417 |
| 2015 | 28 | 1.36874 | 138 | 1.11922 | 754 | 2.81955 | 5,007 | 1.94123 |
| 2016 | 38 | 1.88622 | 183 | 1.42339 | 808 | 2.91014 | 5,029 | 1.93391 |
| 2017 | 28 | 1.34304 | 183 | 1.30779 | 901 | 3.06073 | 5,219 | 1.96466 |
| 2018 | 39 | 1.54558 | 205 | 1.42316 | 918 | 3.04887 | 5,727 | 2.08690 |
| 2019 | 43 | 1.64141 | 238 | 1.51619 | 924 | 3.03584 | 5,855 | 2.11627 |
| 2020 | 48 | 1.74988 | 252 | 1.53300 | 1,157 | 3.60940 | 6,350 | 2.29218 |

*AIAN indicates American Indian or Alaska Native; API, Asian or Pacific Islander; AAMR, age-adjusted mortality rate per 100,000 population. Annual deaths among American Indian or Alaska Native individuals ranged from 15 to 48; the corresponding Joinpoint estimates are exploratory because sparse annual counts produced wide confidence intervals and a short three-year segment.*

**Supplementary Table 5. Annual mortality counts and age-adjusted mortality rates by Hispanic origin, 1999–2023.**

| **Year** | **Hispanic** | | **Non-Hispanic** | | **Not stated** | |
| --- | --- | --- | --- | --- | --- | --- |
|  | **Deaths** | **AAMR** | **Deaths** | **AAMR** | **Deaths** | **AAMR** |
| 1999 | 224 | 2.63496 | 8,459 | 4.12976 | 24 | Not Applicable |
| 2000 | 223 | 2.45316 | 8,072 | 3.88928 | 22 | Not Applicable |
| 2001 | 260 | 2.61160 | 7,647 | 3.63583 | 23 | Not Applicable |
| 2002 | 232 | 2.21895 | 7,632 | 3.58179 | 11 | Not Applicable |
| 2003 | 264 | 2.48616 | 7,468 | 3.48104 | 21 | Not Applicable |
| 2004 | 230 | 1.96685 | 6,762 | 3.08892 | 10 | Not Applicable |
| 2005 | 257 | 2.10220 | 6,495 | 2.89549 | Suppressed | Not Applicable |
| 2006 | 240 | 1.85547 | 5,862 | 2.56448 | Suppressed | Not Applicable |
| 2007 | 247 | 1.78939 | 5,533 | 2.38597 | Suppressed | Not Applicable |
| 2008 | 254 | 1.76212 | 5,365 | 2.26234 | 11 | Not Applicable |
| 2009 | 265 | 1.73661 | 4,954 | 2.03678 | 11 | Not Applicable |
| 2010 | 244 | 1.55024 | 5,041 | 2.06589 | Suppressed | Not Applicable |
| 2011 | 273 | 1.58062 | 5,088 | 2.00620 | Suppressed | Not Applicable |
| 2012 | 257 | 1.41887 | 4,988 | 1.91842 | 15 | Not Applicable |
| 2013 | 289 | 1.46714 | 5,149 | 1.94551 | 13 | Not Applicable |
| 2014 | 319 | 1.55008 | 5,224 | 1.92871 | 13 | Not Applicable |
| 2015 | 383 | 1.71566 | 5,526 | 2.02433 | 18 | Not Applicable |
| 2016 | 381 | 1.63034 | 5,665 | 2.05173 | 12 | Not Applicable |
| 2017 | 406 | 1.59155 | 5,908 | 2.08585 | 17 | Not Applicable |
| 2018 | 456 | 1.72693 | 6,421 | 2.20897 | 12 | Not Applicable |
| 2019 | 479 | 1.71644 | 6,571 | 2.23988 | 10 | Not Applicable |
| 2020 | 532 | 1.88168 | 7,260 | 2.42272 | 15 | Not Applicable |
| 2021 | 575 | 1.97136 | 7,695 | 2.72163 | 11 | Not Applicable |
| 2022 | 596 | 1.94209 | 8,312 | 2.75950 | 10 | Not Applicable |
| 2023 | 622 | 2.01447 | 8,367 | 2.79433 | 22 | Not Applicable |

*AAMR indicates age-adjusted mortality rate per 100,000 population. 'Not Applicable' is reproduced exactly from the source file.*

**Supplementary Table 6. Age-adjusted mortality rates by urbanization category, 1999–2020.**

| **Urbanization category** | **AAMR** | **Lower 95% CI** | **Upper 95% CI** | **SE** |
| --- | --- | --- | --- | --- |
| Large Central Metro | 2.22369 | 2.20042 | 2.24697 | 0.01188 |
| Large Fringe Metro | 2.09890 | 2.07445 | 2.12335 | 0.01247 |
| Medium Metro | 2.46967 | 2.44175 | 2.49759 | 0.01424 |
| Small Metro | 2.75673 | 2.71335 | 2.80010 | 0.02213 |
| Micropolitan (Nonmetro) | 3.17184 | 3.12540 | 3.21828 | 0.02369 |
| NonCore (Nonmetro) | 3.40654 | 3.35278 | 3.46030 | 0.02743 |

*AAMR indicates age-adjusted mortality rate per 100,000 population.*

**Supplementary Table 7. State-level pooled age-adjusted mortality rates for U.S. deaths with stroke as the underlying cause and heart failure as a contributing condition, 1999–2020.**

| **State** | **AAMR** | **State** | **AAMR** | **State** | **AAMR** |
| --- | --- | --- | --- | --- | --- |
| Alabama | 3.27337 | Alaska | 2.52917 | Arizona | 1.05133 |
| Arkansas | 4.01320 | California | 2.82598 | Colorado | 2.60663 |
| Connecticut | 1.82532 | Delaware | 1.69206 | District of Columbia | 2.14909 |
| Florida | 1.29326 | Georgia | 2.45419 | Hawaii | 1.80712 |
| Idaho | 2.53783 | Illinois | 2.29507 | Indiana | 2.92808 |
| Iowa | 2.50373 | Kansas | 2.40603 | Kentucky | 3.20700 |
| Louisiana | 2.37308 | Maine | 1.83208 | Maryland | 2.21502 |
| Massachusetts | 1.62189 | Michigan | 2.66729 | Minnesota | 3.14193 |
| Mississippi | 5.36190 | Missouri | 2.48638 | Montana | 2.50418 |
| Nebraska | 2.97085 | Nevada | 1.33822 | New Hampshire | 1.88394 |
| New Jersey | 1.66285 | New Mexico | 2.05376 | New York | 1.45469 |
| North Carolina | 2.83741 | North Dakota | 3.66697 | Ohio | 2.99754 |
| Oklahoma | 4.29131 | Oregon | 3.62482 | Pennsylvania | 2.12170 |
| Rhode Island | 2.13878 | South Carolina | 3.00091 | South Dakota | 3.53954 |
| Tennessee | 3.11701 | Texas | 3.41632 | Utah | 2.49719 |
| Vermont | 2.50011 | Virginia | 2.27004 | Washington | 3.26198 |
| West Virginia | 3.60390 | Wisconsin | 2.43978 | Wyoming | 1.98198 |

*AAMR indicates age-adjusted mortality rate per 100,000 population.*

**Supplementary Table 8. Exploratory Joinpoint estimates for deaths with stroke as the underlying cause and heart failure as a contributing condition among American Indian or Alaska Native individuals, 1999–2020.**

| **Measure** | **Period** | **Estimate, %** | **95% CI** | **p-value** |
| --- | --- | --- | --- | --- |
| APC | 1999–2005 | +2.9060 | −3.9588 to +24.8500 | 0.241552 |
| APC | 2005–2008 | −23.4867 | −30.7442 to −6.5384 | 0.035193 |
| APC | 2008–2020 | +2.1458 | −0.5406 to +9.2258 | 0.075585 |
| AAPC | 1999–2020 | −1.7767 | −2.9598 to +0.7951 | 0.111578 |

*Annual deaths ranged from 15 to 48. Because the series contained sparse annual counts, wide segment-level confidence intervals, and a short 2005–2008 segment, these estimates are exploratory and should not be interpreted as evidence of a stable directional trend. AAPC indicates average annual percent change; APC, annual percent change; CI, confidence interval.*

**Supplementary Methods S1. Pre-pandemic sensitivity and robustness analyses.**

The primary pre-pandemic sensitivity analysis restricted the overall annual series to 1999–2019 and was conducted using Joinpoint Regression Program version 5.4.0.0, permitting zero to three joinpoints. The outcome definition, annual age-adjusted mortality rates, CDC WONDER-derived standard errors, logarithmic transformation, uncorrelated-error assumption, Weighted Bayesian Information Criterion model selection, and empirical quantile confidence interval procedure were otherwise identical to those used in the primary 1999–2023 analysis. Robustness analyses were then performed using version 6.0.1 while permitting zero to three and zero to four joinpoints. All three specifications selected the same three joinpoints (2003, 2009, and 2014) and produced identical APC, AAPC, confidence-interval, p-value, fitted-value, SSE, and MSE results. To evaluate the potential influence of serial autocorrelation on the pre-pandemic finding, additional analyses were conducted in Joinpoint version 6.0.1 using first-order autocorrelated [AR(1)] errors while retaining the CDC WONDER-derived standard errors, logarithmic transformation, zero-to-three-joinpoint range, Weighted Bayesian Information Criterion model selection, and empirical quantile confidence interval procedure with 5,001 resamples. The lag-1 autocorrelation coefficient was first estimated from the data and was −0.165. Sensitivity analyses were additionally performed with fixed positive autocorrelation coefficients of ρ = 0.10, 0.20, and 0.30. All AR(1) specifications selected joinpoints in 2003, 2009, and 2014, and the 2014–2019 APC remained statistically significant under every specification (Supplementary Table 11).

**Supplementary Table 9. Pre-pandemic Joinpoint sensitivity analysis for mortality with stroke as the underlying cause and heart failure as a contributing condition, United States, 1999–2019.**

| **Measure** | **Period** | **Estimate, %** | **95% CI** | **p-value** |
| --- | --- | --- | --- | --- |
| APC | 1999–2003 | −4.6458 | −5.5867 to −3.1464 | <0.000001 |
| APC | 2003–2009 | −8.0621 | −9.4678 to −7.4248 | <0.000001 |
| APC | 2009–2014 | −1.2110 | −3.2334 to +0.2239 | 0.068786 |
| APC | 2014–2019 | +3.0377 | +2.0611 to +4.7692 | <0.000001 |
| AAPC | 1999–2019 | −2.9842 | −3.1531 to −2.8075 | <0.000001 |

*Selected joinpoints were 2003 (95% CI, 2002–2005), 2009 (95% CI, 2008–2010), and 2014 (95% CI, 2012–2016). The significant 2014–2019 APC indicates that the late-period increase was evident before the COVID-19 pandemic. APC indicates annual percent change; AAPC, average annual percent change; CI, confidence interval.*

**Supplementary Table 10. Robustness of the pre-pandemic sensitivity analysis across software versions and model-complexity specifications.**

**Panel A. Three-way specification comparison.**

| **Specification** | **Joinpoint version** | **Maximum joinpoints** | **Selected joinpoints** | **Terminal APC (95% CI), %** | **Full-period AAPC (95% CI), %** |
| --- | --- | --- | --- | --- | --- |
| Primary sensitivity analysis | 5.4.0.0 | 3 | 2003, 2009, 2014 | +3.0377 (+2.0611 to +4.7692) | −2.9842 (−3.1531 to −2.8075) |
| Updated software check | 6.0.1 | 3 | 2003, 2009, 2014 | +3.0377 (+2.0611 to +4.7692) | −2.9842 (−3.1531 to −2.8075) |
| Expanded candidate-range check | 6.0.1 | 4 | 2003, 2009, 2014 | +3.0377 (+2.0611 to +4.7692) | −2.9842 (−3.1531 to −2.8075) |

**Panel B. Candidate-model comparison in the expanded 0–4 joinpoint analysis.**

| **Candidate model** | **SSE** | **BIC** | **Weighted BIC** | **Selected** |
| --- | --- | --- | --- | --- |
| 3 joinpoints | 27.4103 | 1.4262 | 1.8336 | Yes |
| 4 joinpoints | 23.3768 | 1.5570 | 2.0397 | No |

*The selected model and substantive conclusions were unchanged across all three specifications. In the expanded 0–4 joinpoint analysis, the four-joinpoint candidate reduced SSE but had higher BIC and Weighted BIC values; therefore, the more parsimonious three-joinpoint model remained selected. The terminal APC and full-period AAPC were statistically significant at p<0.000001 in all specifications.*

**Supplementary Table 11. Autocorrelation-robust sensitivity analyses of the pre-pandemic mortality trend, United States, 1999–2019.**

| **Error specification** | **ρ** | **Selected joinpoints** | **Terminal APC (95% CI), %** | **p-value** | **Full-period AAPC (95% CI), %** | **p-value** |
| --- | --- | --- | --- | --- | --- | --- |
| Uncorrelated, primary sensitivity analysis | — | 2003, 2009, 2014 | +3.0377 (+2.0611 to +4.7692) | <0.000001 | −2.9842 (−3.1531 to −2.8075) | <0.000001 |
| AR(1), estimated from data | −0.165 | 2003, 2009, 2014 | +3.0442 (+2.1091 to +4.6983) | <0.000001 | −2.9801 (−3.1436 to −2.8039) | <0.000001 |
| AR(1), fixed | +0.10 | 2003, 2009, 2014 | +3.0325 (+1.7442 to +6.4909) | 0.003599 | −2.9867 (−3.2027 to −2.7438) | <0.000001 |
| AR(1), fixed | +0.20 | 2003, 2009, 2014 | +3.0262 (+1.5207 to +7.0457) | 0.005599 | −2.9892 (−3.2488 to −2.7050) | <0.000001 |
| AR(1), fixed | +0.30 | 2003, 2009, 2014 | +3.0187 (+1.0078 to +8.0227) | 0.019996 | −2.9916 (−3.3387 to −2.6378) | <0.000001 |

*APC indicates annual percent change; AAPC, average annual percent change; AR(1), first-order autoregressive error structure; CI, confidence interval. All AR(1) analyses used Joinpoint Regression Program version 6.0.1, CDC WONDER-derived standard errors, logarithmic transformation, a zero-to-three-joinpoint range, Weighted Bayesian Information Criterion model selection, and empirical quantile confidence intervals with 5,001 resamples. All specifications retained the 2003, 2009, and 2014 joinpoints. The significant 2014–2019 increase therefore remained robust across the tested serial-autocorrelation assumptions.*

**Supplementary Figure S1.**

**Supplementary Figure S1. Pre-pandemic Joinpoint analysis of age-adjusted mortality rates for deaths with stroke as the underlying cause and heart failure as a contributing condition, United States, 1999–2019. Joinpoints were identified in 2003, 2009, and 2014. AAMR increased significantly from 2014 to 2019 (APC, +3.04%; 95% CI, +2.06% to +4.77%; p<0.000001). Asterisks indicate APCs significantly different from zero at α=0.05.**
